# Ferroptosis as a potential target for cancer therapy

**DOI:** 10.1038/s41419-023-05930-w

**Published:** 2023-07-24

**Authors:** Zhen Chen, Weilong Wang, Siti Razila Abdul Razak, Tao Han, Nor Hazwani Ahmad, Xiumin Li

**Affiliations:** 1 Department of Gastroenterology, the Third Affiliated Hospital of Xinxiang Medical University, Henan Key Laboratory of Tumor Molecular Therapy Medicine, Xinxiang, 453003 Henan Province China; 2 Department of Biomedical Science, Advanced Medical and Dental Institute, Universiti Sains Malaysia, Bertam 13200, Kepala Batas, Pulau Pinang Malaysia; 3 Xinxiang Key Laboratory for Molecular Therapy of Cancer, Xinxiang Medical University, Xinxiang, 453003 Henan Province P. R. China; 4 Institutes of Health Central Plains, Xinxiang Medical University, Xinxiang, 453003 Henan Province P. R. China

**Keywords:** Cancer therapy, Cancer, Cancer metabolism, Cancer microenvironment

## Abstract

Ferroptosis is a recently discovered essential type of cell death that is mainly characterized by iron overload and lipid peroxidation. Emerging evidence suggests that ferroptosis is a double-edged sword in human cancer. However, the precise underlying molecular mechanisms and their differential roles in tumorigenesis are unclear. Therefore, in this review, we summarize and briefly present the key pathways of ferroptosis, paying special attention to the regulation of ferroptosis as well as its dual role as an oncogenic and as a tumor suppressor event in various human cancers. Moreover, multiple pharmacological ferroptosis activators are summarized, and the prospect of targeting ferroptosis in cancer therapy is further elucidated.

## Facts

1. Ferroptosis often dysregulated in human cancer.
2. Ferroptosis plays a dual role in cancer.
3. Ferroptosis provides a promising strategy for cancer therapy.

## Open questions

1. Why ferroptosis is a double-edged sword in cancer and what is its mechanism?
2. What is the mechanism of ncRNAs regulating ferroptosis and whether they can be the targets of ferroptosis?
3. In clinical treatment, how to combine ferroptosis inducers with anti-tumor therapy to achieve optimal therapeutic effect?

## Introduction

**Fig. 1:**
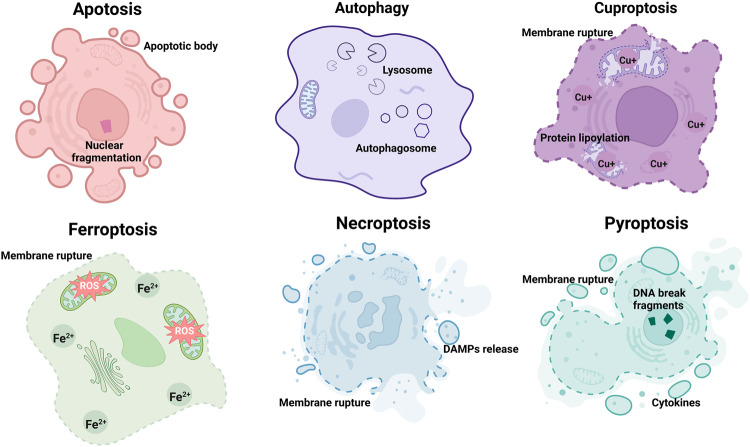
Morphological and biochemical characteristics of apoptosis, autophagy, cuproptosis, ferroptosis, necroptosis, and pyroptosis. ROS reactive oxygen species, DAMPs damage-associated molecular patterns, Cu^+^ cuprous ion, Fe^2+^ ferrous ion.

**Fig. 2:**
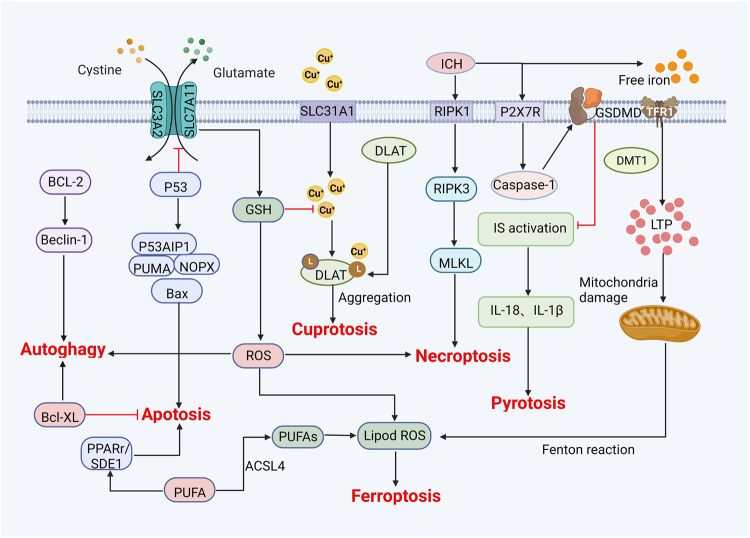
Metabolic pathways of ferroptosis and crosstalk with other types of cell death mechanisms. BCL-2 B-cell lymphoma-2, Bcl-XL BCL2 like 1, PUFA polyunsaturated fatty acids, GSH glutathione, ROS reactive oxygen species, ACSL4 acyl-CoA synthetase long-chain family member 4, DLAT recombinant dihydrolipoyl transacetylase, ICH intracerebral hemorrhage, RIPK1/3 receptor-interacting serine/threonine protein kinases1/3, MLKL mixed lineage kinase domain-like protein, P2X7R P2x7 receptor, IL-18 interleukin-18, IL-1β interleukin-1beta, GSDMD Gasdermin D, TFR1 transferrin receptor 1, DMT1 divalent metal transporter 1, LIP labile iron pool, Cu^+^ cuprous ion.

In 2020, there were an estimated 19.3 million new cancer cases and nearly 10 million mortalities due to cancer worldwide [1]. Despite significant advances in treatment, cancer remains the leading cause of death in humans. Furthermore, toxic effects and drug resistance of traditional therapies pose major challenges. As a result, the search for more effective and tolerable anticancer therapies continues [2]. Ferroptosis was first defined by Brent R. Stockwell in 2012 as an iron-dependent type of cell death that is driven by lipid reactive oxygen species (ROS) [3–5] and is significantly different from other regulated cell death forms such as apoptosis [6, 7], autophagy [8, 9], cuproptosis [10, 11], necroptosis [12, 13], and pyroptosis [13, 14] in terms of cell morphology, biochemistry, and genetics (Fig. 1, Fig. 2 and Table 1).

**Table 1:** Main features of apoptosis, autophagy, cuproptosis, ferroptosis, necroptosis and pyroptosis.

Type of cell death	Morphological features	Biochemical features	Key regulators	Reference
Apoptosis	The permeability of cell membrane is enhanced, chromatin condensation, nuclear fragmentation, cell membrane blistering, cell shrinkage, the formation of apoptotic bodies	Pro-apoptotic BCL-2 protein family members, activation of caspases, oligonucleosomal DNA fragmentation	BCL-2, P53, Caspase, FAS, BAX, BAK, BCL-XL	[6, 7]
Autophagy	Accumulation of autophagic vacuoles, vacuolisation of the cytoplasm, formation of double-membraned, autolysosomes, no chromatin condensation and lack of change in the plasma membrane	Conversion of LC3-I to LC3-II, Increase of lysosomal activity and degradation of P62	ATG5, ATG7, Beclin 1, TFEB	[8, 9]
Cuproptosis	Rupture of plasma membrane, other unknown	Copper accumulation, protein lipoylation	FDX1, LIAS, LIPT1, DLAT, GLS, MTF	[10, 11]
Ferroptosis	Cell membrane rupture and vesicle, increase of cell membrane density, atrophy of mitochondria, reduction or even disappearance of mitochondrial ridge, chromatin condensation	Iron-overload, accumulation of ROS, Inhibition of system XC— and depletion of GSH, release of arachidonic acid mediators	SLC7A11, GPX4, P53, VDAC2/3, NRF2, LSH, TFR1, NOX	[3–5]
Necroptosis	Rupture of plasma membrane, swelling of cytoplasmic, chromatin condensation(moderate)	Decrease in ATP level, activation of RIP1, RIP3, and MLKL, PARP1 hyperactivation and release of DAMPs	LEF1, RIP1/3, MLKL	[12, 13]
Pyroptosis	Rupture of plasma membrane, karyopyknosis, cytoplasmic swelling and DNA breaks	Activation of caspase-1 and the release of inflammatory cytokines IL-1β and IL-18	CASP1/4, IL-1, IL-18, GSDMD	[13, 14]

*BCL-2* B-cell lymphoma-2, BAX BCL2 associated X, apoptosis regulator, *BAK* BCL2 antagonist/killer 1, *BCL-XL* BCL2 like 1, *ATG5* autophagy related gene 5, *ATG7* autophagy related gene 7, *TFEB* transcription factor EB, *FDX1* ferredoxin 1, *LIAS* lipoic acid synthetase, *LIPT1* lipoyltransferase 1, *DLAT* dihydrolipoyl transacetylase, *GLS* glutaminase, *MTF1* metal regulatory transcription factor 1, *SLC7A11* solute carrier family 7 member 11, *GPX4* glutathione peroxidase 4, *VDAC2/3* voltage dependent anion channel 2/3, *NRF2* nuclear factor erythroid 2-related factor 2, *LSH* lymphocyte stimulating hormone, *TFR1* transferrin receptor, *NOX* NADPH oxidase, *LEF1* lymphoid enhancer binding factor 1, *RIP1/3* receptor interacting protein kinase1/3, *MLKL* mixed lineage kinase domain-like, *CASP1/4* caspase 1/4, *IL-1* interleukin-1, *IL-18* interleukin-18, *GSDMD* Gasdermin D.

Both the extrinsic pathway and the intrinsic pathway play a crucial role in this process. On the one hand, the extrinsic pathway is activation by inhibition of the cystine-glutamate antiporter (system X_C_^—^). The absorption of extracellular cystine is hindered by the decreased activity of system X_C_^—^, which subsequently affects the synthesis of glutathione (GSH) [15]. This reduction in the cell’s antioxidant capacity can result in the accumulation of lipid ROS, thus promoting ferroptosis [15, 16]. In contrast, activation of iron transporters such as serum transferrin and lactotransferrin could trigger the extrinsic pathway. By increasing iron intake and limiting iron loss, these transporters induce ferroptosis via enhancing iron accumulation [15, 17]. The intrinsic pathway is realized by blocking the activation of intracellular antioxidant enzymes. For example, GPX4 can reduce the cytotoxic lipid peroxide (L-OOH) to the corresponding alcohol (L-OH). Once the activity of GPX4 is inhibited, it will lead to the accumulation of lipid peroxide in the cell membrane [15, 18]. In addition, long-chain fatty acid coenzyme A ligase 4 (ACSL4) encourage the incorporation of polyunsaturated fatty acids (PUFA) into phospholipids to form polyunsaturated fatty acid phospholipids (PUFA-PL). PUFA-PLs are susceptible to oxidation induced by free radicals mediated by lipoxygenases (ALOXs), which eventually lead to the destruction of the lipid bilayer and affect the membrane function, thus triggering ferroptosis [19, 20]. Both extrinsic and intrinsic pathways can be directly activated by specific small molecules, such as RAS-selective lethal (RSL3) and erastin. Therefore, the selection of small molecules for extrinsic or intrinsic pathways can be based on the level of development difficulty. However, some small molecules such as ferristatin 1 are not suitable for in vivo experiments due to its solubility, stability, pharmacokinetics, and drug delivery route, which is a major challenge in clinical transformation.

Recently, ferroptosis has been shown to be involved in different diseases including neurodegeneration and malignant tumors such as liver cancer, breast cancer, and lung cancer [21–24]. Over the past two decades, ferroptosis has been extensively studied in a variety of malignancies, and it has been shown to regulate cancer progression through various signaling pathways. Fascinatingly, it is not as only a tumor suppressor, but it also exerts oncogenic function. Of note, cancer cells that are resistant to traditional therapies or have a high propensity to metastasize are more prone to ferroptosis [25, 26]. Hence, the regulation of ferroptosis and its target proteins is considered a novel and promising cancer therapy strategy. The multiple signaling pathways involved in ferroptosis have been well summarized elsewhere [27, 28]. Herein, the purpose of this article is to provide insights into the involvement of ferroptosis, along with its regulators and promoters, in tumor development and as a potential treatment target. In particular, potential applications of ferroptosis in chemotherapy, radiotherapy, immunotherapy, nanotherapy, sonodynamic therapy (SDT) and photodynamic therapy (PDT) are discussed, with an emphasis on potential therapeutic agents for clinical applications.

## Ferroptosis is an essential type of cell death

Ferroptosis is defined as a form of regulated cell death that is characterized by the accumulation of iron and ROS [3–5]. Specifically, ROS is highly active oxidation radical, including superoxides, peroxides and free radicals [29, 30]. Fenton reaction is an important source of ROS, which is produced by the interaction of ferrous iron ion and hydrogen peroxide in cells through Fenton reaction [30, 31]. Subsequently, ROS reacts with PUFAs to deprive the hydrogen atom between its long chain double bonds, causing lipid peroxidation and ultimately leading to ferroptosis [31, 32]. On the other hand, mitochondria are another essential source of ROS. During oxidative phosphorylation of mitochondria, ROS is produced after the combination of electrons and molecular oxygen, which increases the oxidative stress response of cells, accompanied by lipid, protein, and DNA damage [31–33]. This will lead to accumulation of lipid ROS, atrophy of mitochondria, increase of cell membrane density, and eventually result in ferroptosis. Interestingly, the signaling mechanisms of ferroptosis and other cell death types of crosstalk with each other. For example, ROS is essential for autophagy, apoptosis, and ferroptosis; in addition, the core regulator of ferroptosis, GSH, is also involved in the regulation of cuproptosis [34, 35]. Nonetheless, ferroptosis has been reported to induce specific cellular morphological and biochemical changes and to be regulated by multiple metabolic pathways (Figs. 1, 2 and Table 1).

### Morphological and biochemical features of ferroptosis

The morphological features of cells undergoing ferroptosis differ from those of cells undergoing autophagy or apoptosis. Specifically, the cell membrane is not damaged during apoptosis or autophagy; while in ferroptosis, the cell membrane density is increased and ruptured, leading to the formation of vesicles. Additionally, the hallmarks of necroptosis or pyroptosis are cytoplasmic swelling and DNA breakage, respectively [13, 36]. However, in ferroptosis, the distinctive characteristics are manifested as mitochondrial atrophy as well as a reduction or even disappearance of the mitochondrial ridge, but there is no change in the size of the nucleus except for chromatin condensation [24, 37, 38]. Moreover, the signs of apoptosis, pyroptosis, and cuprotosis are the formation of apoptotic bodies, the release of inflammatory cytokines, and copper accumulation; while ferroptosis is mainly distinguished by an excess of iron and ROS [3–5, 10, 39, 40]. In short, ferroptosis and known forms of regulated cell death have distinct morphological and biochemical features (Fig. 1 and Table 1).

### Metabolic changes in ferroptosis

#### Iron metabolism imbalance

Iron metabolism is regulated by multiple steps, including iron absorption, utilization, recycling, and storage. Disruption of iron metabolism leads to excessive accumulation of intracellular iron, which causes the generation of free radicals and oxidative stress [41, 42]. Specifically, as one of the essential elements in the proliferation and development of tumor cells, iron is also the core element for ferroptosis [43, 44]. In addition to its role in the production of DNA and ATP, iron is a crucial component of the mitochondrial electron transport chain and a cofactor of metalloproteinases. For example, ferrithioprotein is simultaneously a vital cofactor of oxidoreductases in the mitochondrial electron transport chain and a cofactor of many vital enzymes in redox reactions. Extracellular ferric ions (Fe^3+^) combine with transferrin to form the transferrin–Fe^3+^ complex, which enters the cell mediated by the membrane protein transferrin receptor 1 and is reduced to Fe^2+^. With the assistance of the divalent metal transporter 1 (solute carrier family 11 member 2; SLC11A2) or Zrt- and Irt-like proteins 8 and 14 (SLC39A8 and SLC39A14, respectively), the labile iron pool stores Fe^2+^ in the cell [43, 45, 46]. Meanwhile, Fe^2+^, with the help of iron chaperones such as poly(rC)-binding proteins 1 and 2, pumps iron through membrane ferroportin (FPN) 1 to maintain the balance of intracellular iron [43, 47]. However, once Fe^2+^ in cells is overloaded, the Fenton reaction will occur with hydrogen peroxide, resulting in excessive ROS and driving ferroptosis (Fig. 2).

#### Lipid metabolism disorder

Lipids have crucial roles in energy storage, signal transmission, membrane development, cell membrane formation, energy storage, and signal transduction. Lipid metabolism regulates cell lipid toxicity, and abnormal lipid metabolism is considered a hallmark of malignancy and an essential factor for ferroptosis [48]. Additionally, the metabolism of lipids in cells relies heavily on fatty acids. According to their saturation levels, fatty acids are divided into three categories: saturated fatty acids, PUFAs, and monounsaturated fatty acids (MUFAs). Among them, PUFAs and MUFAs have been demonstrated to contribute to ferroptosis [49, 50]. Due to the weak C–H bond at the diallyl position, PUFAs on the cell membrane are vulnerable to attack by ROS, which can induce lipid peroxidation [51]. In this process, acyl-CoA synthetase long-chain family member 4 (ACSL4) is required for the production of PUFAs, thereby positively regulating ferroptosis [51]. In contrast, exogenous MUFAs such as exogenous palmitic acid and oleic acid have been reported to negatively regulate drug-induced ferroptosis [52, 53]. Exogenous MUFAs can be activated by acyl-CoA synthetase long-chain family member 3 to displace PUFAs at the plasma membrane and to reduce the sensitivity of plasma membrane lipids to oxidation [53]. Moreover, an increased ratio of MUFAs to PUFAs can be observed on cancer cell membranes, thereby inhibiting lipotoxicity and ferroptosis [54] (Fig. 2).

#### Amino acid metabolism dysfunction

Amino acids are essential nutrients for the survival of cells and are involved in deamination, decarboxylation, ammonia metabolism, and oxidative decomposition capacity metabolic processes. Meanwhile, abnormal amino acid metabolism leads to redox imbalance, energy regulation disorder, and biosynthesis dysfunction, thus providing support for tumor proliferation [55]. Ferroptosis induced by an abnormal amino acid metabolism is mainly related to GSH. GSH is a tripeptide compound composed of glutamate, cysteine, and glycine (γ-glutamyl-L-cysteinyl-L-glycine) and is an important antioxidant and free radical scavenger in the body [56, 57]. System X_C_^—^ and GPX4 are key regulators of GSH biosynthesis and degradation. System X_C_^—^ is composed of light (SLC7A11) and heavy chain (SLC3A2) subunits, which play an essential role in maintaining the balance of GSH in cells [58, 59]. System X_C_^—^ facilitates the interchange of cystine and glutamate across the plasma membrane, controls GSH production in response to extracellular glutamate levels, and transports glutamate from inside the cell to outside the cell [60]. Impaired function of system X_C_^—^ or insufficient intracellular cysteine levels can lead to the decreased synthesis of GSH, triggering ferroptosis. On the other hand, as an important enzyme for scavenging lipid oxygen free radicals, GPX4 can use GSH as a substrate to reduce membrane lipid hydrogen peroxide to nontoxic lipid alcohols, decrease oxidative stress damage, and negatively regulate ferroptosis [56, 57] (Fig. 2).

## Regulation of ferroptosis in cancer

Ferroptosis can be regulated by a variety of mechanisms, especially by the transcription of genes and post-translation of proteins.

### Noncoding RNAs (ncRNAs) promote ferroptosis in cancer

Numerous studies have demonstrated that ncRNAs can promote ferroptosis in multiple cancers (Table 2). For example, microRNA (miRNA or miR)-15a and miR-15a-3p have been reported to target GPX4 to promote ferroptosis in prostate cancer and colorectal cancer, respectively [61, 62]. Furthermore, GPX4 inhibition triggers the sensitivity of ferroptosis by restoring the miR-4715-3p levels, which are suppressed in upper gastrointestinal cancers [63]. A similar mechanism has been observed in non-small cell lung cancer. miR-324-3p causes cisplatin resistance by suppressing GPX4 expression [64], while miR-302a-3p acts as a positive regulator of ferroptosis by targeting FPN [65]. In addition to inducing ferroptosis via GPX4, a study by Bai et al. has shown that miR-214-3p the GSH axis in hepatoma and thus acts as a tumor inhibitor [66].

**Table 2:** Noncoding RNAs that promote ferroptosis in cancer.

ncRNAs	Targets	Model	Mechanism summary	Reference
miR-15a	GPX4	LNCaP cell line	miR-15a promotes ferroptosis by inhibiting GPX4 expression.	[61]
miR-15a-3p	GPX4	Patient’s tumor tissues; nude mice; HCT‐116, CaCo2, HT29, and KM12 cell lines	miR-15a-3p positively regulates ferroptosis by directly targeting GPX4, thereby inhibiting cancer cell proliferation, migration, and invasion.	[62]
miR-4715-3p	AURKA/ GPX4	Patient’s tumor tissues; MKN45 and STKM2 cell lines	miR-4715-3p mediates decreased AURKA levels, and inhibition of AURKA or recombination of miR-4715-3p inhibits GPX4 and induces ferroptosis.	[63]
miR-324-3p	GPX4	A549 cell line	miR-324-3p directly targets GPX4 to induce ferroptosis and reverse cisplatin resistance.	[64]
miR-302a-3p	FPN	A549, H358, H1299, and H1650 cell lines	miR-302a-3p increases ROS accumulation, induces ferroptosis, and inhibits cell growth by targeting FPN.	[65]
miR-214-3p	ATF4/GSH	nude mice; HepG2 and Hep3B cell lines	miR-214 directly targets ATF4, reduces GSH, enhances erastin-induced ferroptosis, and suppresses tumor growth.	[66]
lncPVT1	miR-214-3p/GPX4	Patient’s tumor tissues; nude mice; HepG2 and Huh7 cell lines	Depletion of lncPVT1 accelerates ferroptosis through the miR-214-3p/GPX4 axis.	[67]
SLC16A1-AS1	miR-1433p/SLC7A11	HK-2, 786-O, A498, and Caki-1 cell lines	lncRNA SLC16A1-AS1 induces ferroptosis and inhibits cell viability, proliferation, and migration through miR-143-3p/SLC7A11 signaling	[68]
LINC00618	LSH/SLC7A11	HL60, K562, MV4-11 and CCRF- CEM cell lines	LINC0618 interacts with LSH to affect the expression of SLC7A11 and trigger ferroptosis.	[69]
ARHGEF26-AS1	miR-372-3p/ ADAM23/GPX4/SLC7A11	Ec9706, TE-1, and EC109 cell lines	ARHGEF26-AS1 promotes ferroptosis by inhibiting SLC7A11 but inhibits cell growth via miR-372-3p.	[70]
P53RRA	G3BP1/P53	A549, H358, and H522 cell lines	P53RRA induces ferroptosis and acts as a potential tumor suppressor by displacing p53 from the cytosolic G3BP1 complex.	[71]
MT1DP	miR-365a-3p/NRF2	Patient’s tumor tissues; nude mice; A549 and H1299 cell lines	MT1DP sensitizes ferroptosis by upregulating MDA and ROS levels, and reducing GSH levels via miR-365a-3p/NRF2 axis.	[72]
circ0000190	miR-382-5p/ ZNRF3	Patient’s tumor tissues; AGS, KATO III, MKN1, and HGC27 cell lines	circ_0000190 sponges miR-382-5p, inhibits cell proliferation, and promotes erastin-induced ferroptosis by targeting ZNRF3.	[73]
circ0007142	miR-874-3p	Patient’s tumor tissues; nude mice; HCT116, SW620, and SW480 cell lines	circ_0007142 serves as an miR-874-3p sponge to increase Fe^2+^ levels, activates ferroptosis, and suppresses cancer progression.	[74]
circGFRA1	miR1228/AIFM2/GPX4	Patient’s tumor tissues; nude mice; SKBR3 and BT474 cell lines	Silencing circGFRA1 downregulates GPX4 expression and enhances ferroptosis through the miR-1228/AIFM2 axis.	[75]
circLMO1	miR-4291/ACSL4	Patient’s tumor tissues; SiHa, HeLa, CaSki, and C33A cell lines	circLMO1 suppresses cervical cancer growth and metastasis by triggering miR-4291/ACSL4-mediated ferroptosis.	[76]
circ-IARS	ALKBH5	HepG2, SMMC-7721, and Huh7 cell lines	circ-IARS increases MDA and Fe^2+^ levels and positively regulates ferroptosis through ALKBH5-mediated autophagy.	[77]

*ACSL4* acyl-CoA synthetase long-chain family member 4, *ADAM23* disintegrin and metalloproteinase domain-containing protein 23, *AIFM2* apoptosis-inducing factor mitochondria-associated 2, *ALKBH5* AlkB homolog 5, *ATF4* activating transcription factor 4, *AURKA* Aurora kinase A, *circGFRA1* circular RNA glial cell line-derived neurotrophic factor family receptor alpha-1, *circ-IARS* circular RNA IARS, *circLMO1* circular RNA LIM domain only 1, *FPN* ferroportin, *GPX4* glutathione peroxidase 4, *GSH* glutathione, *G3BP1* GTPase-activating protein-binding protein 1, *LINC00618* long intergenic nonprotein-coding RNA 618, *lncRNA* long noncoding RNA, *lncPVT1* long noncoding RNA plasmacytoma variant translocation 1, *LSH* lymphoid-specific helicase, *MDA* malondialdehyde, *miR* microRNA, *NRF2* nuclear factor erythroid 2-related factor 2, *P53RRA* cytosolic p53-related lncRNA, *ROS* reactive oxygen species, *SLC7A11* solute carrier family 7 member 11, *ZNRF3* zinc and ring finger 3.

Similar to miRNAs, ferroptosis can be accelerated by some long noncoding RNAs (lncRNAs) in various cancers. Taking liver cancer as an example, it has been observed that knockdown of the lncRNA plasmacytoma variant translocation 1 significantly increased the levels of ROS and Fe^2+^, followed by inhibition of cell viability [67]. In renal cancer, silencing of SLC16A1-AS1 reduced the expression of SLC7A11 and significantly decreased the GSH/glutathione disulfide (GSSG) ratio in cells [68]. Similar results were also confirmed by Wang et al., who demonstrated that the long intergenic nonprotein-coding RNA 618 activates ferroptosis via upregulating SLC7A11 and downregulating ACSL4 in acute myeloid leukemia [69]. Mechanistically, the lncRNA ARHGEF26-AS1 acts as a sponge for miR-372-3p, not only promoting ferroptosis but also inhibiting the proliferation and migration of esophageal squamous cell carcinoma cells [70]. Consistently, the cytosolic p53-related lncRNA induces ferroptosis via promoting ROS and intracellular iron accumulation, thereby suppressing lung cancer progression [71]. Additionally, bioinformatics analysis and preclinical experiments have verified that metallothionein 1D, pseudogene enhances erastin-induced ferroptosis by inhibiting nuclear factor erythroid 2-related factor 2 (NRF2) in nonsmall cell lung cancer [72].

A number of circular RNAs also have been reported to be involved in the promotion of ferroptosis. Specifically, according to research by Jiang et al., circ0000190 overexpression accelerated ferroptosis in gastric cancer cells by increasing the levels of malondialdehyde, lipid ROS, and Fe^2+^ [73]. In addition, in colorectal cancer, after knockdown of circ0007142, cells sent out signals for ferroptosis and growth inhibition was observed [74]. Moreover, a preclinical study has shown that apoptosis-inducing factor mitochondria-associated 2 and GPX4 expression as well as the GSH/GSSG ratio were upregulated after knockdown of the circular RNA glial cell line-derived neurotrophic factor family receptor alpha-1 (circGFRA1), suggesting that circGFRA1 promotes ferroptosis via two independent pathways in breast cancer [75]. Of note, experiments performed in vitro and in vivo have revealed that the circular RNA LIM domain only 1 enhances ferroptosis by increasing the expression of ACSL4, which in turn slows down the growth and metastasis of cervical cancer cells [76]. Furthermore, according to RNA-sequencing analysis, the circular RNA IARS (circ-IARS) is overexpressed in hepatocellular carcinoma (HCC), while an in-depth study has found that circ-IARS-silenced cells show a substantial decrease in Fe^2+^ and an evident increase in intracellular GSH. Therefore, it can be speculated that circ-IARS may act as a catalyst for ferroptosis in HCC cells [77].

However, the relationship between ferroptosis and ncRNAs is largely unknown and there are facing many challenges. For example, more detailed and evidence are needed to elucidate the underlying regulatory mechanism between ferroptosis and ncRNAs in the future study. To date, there is still no evidence showing that ncRNAs involved in the occurrence and prognosis of cancer by directly binding to ferroptosis. Therefore, it is essential to further explore more roles of ferroptosis-related ncRNAs in different cancers. In addition, only a fraction of ferroptosis-related ncRNAs have been validated in vivo. Further investigation in large-scale human tissue samples is obviously warranted to determine whether these ncRNAs can be used as a target for clinical.

### Two sides of transcriptional regulators in ferroptosis

**Fig. 3:**
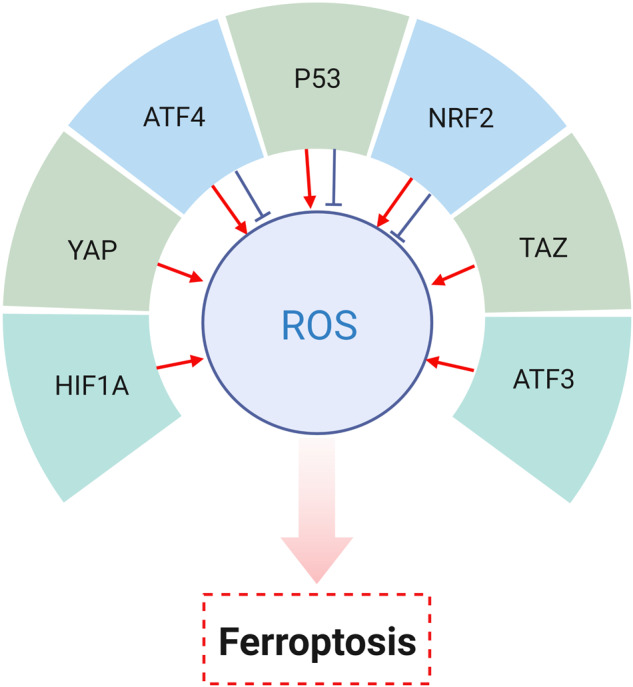
Transcriptional regulators in ferroptosis. ATF activating transcription factor, HIF1A hypoxia-inducible factor 1 alpha, NRF2 nuclear factor erythroid 2-related factor 2, TAZ transcriptional coactivator with PDZ-binding motif, YAP yes-associated protein.

Growing evidence has implicated that transcriptional regulators are double-edged swords in ferroptosis regulation. For example, p53 as a tumor suppressor, has been reported to play a dual role in ferroptosis. In particular, p53 has been demonstrated to induce ferroptosis by directly inhibiting the expression of SLC7A11 and enhancing lipid peroxidase. Of note, it can be detected that the activation of p53 reduced the uptake of cystine, limited intracellular GSH production, thereby activated ferroptosis and suppressed tumor growth [78]. However, p53 also has been proposed to play a role in blocking ferroptosis in human cancer cells. Mechanically, the expression of p53 was increased under the treatment with a small molecule inhibitor, nutlin-3, accompanied by the decrease in GSH consumption and ROS accumulation, ultimately suppressing ferroptosis. Simultaneously, an increase in cell viability can be observed in HT-1080 fibrosarcoma cells [79, 80]. Furthermore, activating transcription factor (ATF) 4 also functions as a negative or positive regulator of ferroptosis in various cancers. On the one hand, ATF4 can drive sorafenib resistance in HCC by inhibiting ferroptosis [81]; on the other hand, sevoflurane has been shown to induce ferroptosis of glioma cells through ATF4 activation [82]. Similarly, ATF3 has been reported to promote ferroptosis and to exert tumor-suppressing effects [83]. Moreover, the abnormal expression of yes-associated protein (YAP) and transcriptional co-activator with PDZ-binding motif (TAZ), two core transcription factors of Hippo pathway, contributes to the cell growth and chemotherapy resistance in multiple cancers [84, 85]. Furthermore, a preclinical study has demonstrated that transcriptional regulatory activity of YAP triggered ferroptosis by targeting transferrin receptor and ACSL4. Briefly, overexpression of YAP lead to an increase of ROS and decrease of cell viability. Moreover, YAP is more susceptible to ferroptosis at high cell density in colon cancer cells [86]. Consistent with this finding, loss of TAZ reduces susceptibility to ferroptosis in multiple cancer cell lines treated with erastin [87]. Besides, hypoxia-inducible factor 1 alpha (HIF1A), as a transcriptional factor of the homeostatic response of cells to hypoxia, is considered to inhibit cancer cell death by promoting lipid accumulation. However, in mice models treated with RSL3, knockout of HIF1A was confirmed to positively regulate ferroptosis by regulating lipid metabolism, thereby effectively inhibiting tumor growth [88, 89]. On the contrary, there is also transcription regulator that promote tumor development by inhibiting ferroptosis. The most direct proof is that NRF2 has been shown to upregulate SLC7A11, thereby protecting tumor cells from ferroptosis [90]. Nevertheless, NRF2 was also reported to trigger ferroptosis via increasing the expression of HMOX1 in lung cancer and renal cell carcinoma (RCC) cells. Specifically, NRF2 can be activated by 4,4'-dimethoxychalcone (DMC) which is extracted from the plant *Angelica keiskei koidzumi*. The activation of NRF2 directly upregulated HMOX1 expression, subsequently led to iron overload and ferroptosis [91, 92] (Fig. 3).

Given a dual role of transcription factors play in ferroptosis, it will be intriguing to further clarify the specific mechanism of this process. It will also be interesting to screen much more transcription factors targeting ferroptosis. In addition, due to the complex regulatory network of ferroptosis, there is uncertainty surrounding the specificity of these transcription factors. Therefore, in-depth studies on both the specificity and preclinical investigations are urgently needed.

### Post-translational modifications in ferroptosis

Ferroptosis plays an important role in the regulation of ubiquitination, phosphorylation, methylation, and acetylation. The key regulatory genes of ferroptosis, such as SLC7A11, GPX4, and voltage-dependent anion-selective channels (VDACs) [1–3], can be regulated by a series of deubiquitinases, including ubiquitin-specific protease (USP) 11 [93], USP14 [94], and OTU domain-containing ubiquitin aldehyde-binding protein 1 [95], as well as ubiquitinases such as neural precursor cell expressed developmentally downregulated protein 4 (NEDD4) [96], NEDD4 ligase [17], and so on. Among the ubiquitinases, NEDD4 is predicted to be the major E3 ligase mediating VDAC1 degradation in melanoma. Another study has confirmed that endogenous VDAC1 interacts with NEDD4, and this contact was enhanced by erastin treatment. However, deletion of VDAC2/3 prevented erastin-induced ferroptosis. Interestingly, these effects were amplified following NEDD4 silencing. Additionally, an in-depth study has revealed that K63, K90, and K163 of the VDAC subtype are critical for NEDD4-mediated ubiquitination [96]. Moreover, ferroptosis activity can be regulated by direct phosphorylation of ACSL4 or associated phosphorylation of SLC7A11. Specifically, protein kinase C βII accelerates ferroptosis by directly phosphorylating ACSL4 at Thr328, which enhances the efficacy of immunotherapy in patients with melanoma [97]. In a preclinical study, phosphorylation of beclin 1 at S90/93/96 contributed to its complexation with SLC7A11 and subsequent lipid peroxidation in ferroptosis, thereby prolonging the survival of mice with pancreatic cancer [98]. In line with the above studies, numerous genes have been reported to regulate ferroptosis by targeting the SLC7A11/GPX4 axis through methylation. Clinically, the high expression of GPX4 may be related to the low level of DNA methylation in multiple cancers. Furthermore, histone H3 lysine-4 trimethylation and acetylation of histone H3 on lysine 27 have been demonstrated to be enriched at the upstream site of GPX4 in different types of cancer tissues, indicating that the high expression of GPX4 may be the result of methylation [99]. An additional study has evaluated the changes in total DNA methylation levels in the multiple myeloma cell lines MM1S and MM1R treated with the GPX4 inhibitor RSL3 and has demonstrated that both MM1S and MM1R converge toward a similar methylation profile under ferroptosis conditions [100]. Besides, a previous study from the Hasegawa group has reported that the ferroptosis inhibitor sulfasalazine, which targets SLC7A11, can increase DNA methylation on the mucin 1 gene (*MUC1*) promoter to regulate *MUC1* gene transcription in triple-negative breast cancer [101]. Moreover, acetylation also has been reported to be involved in the regulation of ferroptosis. For example, Jiang et al. have demonstrated that p53^3KR^ (K117/161/162) regulates SLC7A11 expression and induces ferroptosis [78]. More interestingly, the loss of acetylation at K98 does not affect the transcriptional activity of p53. However, combining the P53^3KR^ and K98 mutations renders P53 utterly incapable of regulating SLC7A11, indicating that K98 acetylation of p53 plays a key role in the inhibition of *SLC7A11* expression and p53-mediated ferroptosis [102]. Nevertheless, regulation of ferroptosis by acetylation requires more research, and the specific mechanism needs to be further elucidated. Therefore, the identification of additional regulators of ferroptosis and acetylation will be necessary for clarifying their roles in cancer.

## Ferroptosis plays a dual role in cancer

### Ferroptosis as a tumor suppressor event

Accumulating evidence indicates that ferroptosis acts as a tumor suppressor and influences the cycle, proliferation, and progression of cancer. So far, in addition to blood-related cancers [103, 104], ferroptosis also acts as a tumor suppressor in multiple solid tumors, including lung cancer [105], breast cancer [106], pancreatic cancer [107], colorectal cancer [108], liver cancer [109], esophageal squamous cell carcinoma [110], gastric cancer [111], and melanoma [112]. Moreover, previous studies have revealed that cytosolic aspartate aminotransaminase (GOT1) is essential for redox balance and the proliferation of pancreatic ductal adenocarcinoma [113]. Recently, suppression of GOT1 has been demonstrated to inhibit GSH biosynthesis by increasing GSSG and NADP^+^, which enhance the effect of butionine sulfoximine (BSO) on cancer cell proliferation. Furthermore, the simultaneous administration of GOT1 and BSO has been shown to significantly slow the progression of xenograft tumors, and GOT1 combined with RSL3 significantly inhibits cell proliferation and enhances cytotoxicity. Interestingly, inhibition of GOT1 combined with RSL3 or erastin can increase lipid ROS accumulation, and this enhancement can be reversed by cotreatment with the lipophilic antioxidant ferrostatin-1 [114].

On the other hand, ferroptosis can cause the migration and invasion of cancer cells to be inhibited. For example, it has been demonstrated that Krüppel-like factor 2 regulates ferroptosis through GPX4, hence preventing cancer cell migration and invasion in clear cell renal cell carcinoma [115]. Another study has revealed that SLC7A11 promotes the migration and invasion of renal cancer cells by enhancing GPX4 export [116]. However, in colorectal cancer cells, GPX4 can be negatively regulated by acyl-coenzyme A dehydrogenase, short/branched chain (ACADSB). The overexpression of ACADSB reduces the migration and invasion of SW620 and LoVo cells by increasing intracellular Fe^2+^ and decreasing the expression of GPX4 and glutathione reductase [117]. In addition to GPX4 and SLC7A11, another key regulator of ferroptosis, ACSL4, has been reported to act as a direct downstream target of miR-211-5p in HCC, and miR-211-5p suppresses malignant phenotypes such as cell proliferation, migration, and invasion by inhibiting ACSL4 expression [118].

Along with ferroptosis blocking cell proliferation, migration, and invasion, an investigation from the Feng group has revealed that ferroptosis is negatively associated with metastasis [119]. They found that lymph node metastases were more common in SLC7A11-positive patients than in SLC7A11-negtive patients, suggesting that SLC7A11 expression is linked to tumor development and metastasis in esophageal squamous cell carcinoma [119]. Furthermore, SLC7A11 overexpression in human HCC is strongly linked with worse tumor differentiation, a higher tumor-nodule-metastasis stage, as well as a poor prognosis [120]. Additionally, Liu et al. have demonstrated that ferroptosis resistance increase the probability of metastasis and have further confirmed that 27-hydroxycholesterol (27HC) is an abundant circulating cholesterol metabolite that can be primarily used to screen cells for increased lipid biosynthesis. The enhanced tumorigenic and metastatic activities of 27HC-resistant cells can be reversed by GPX4 inhibition [121]. Similarly, neratinib, a potent irreversible pan-tyrosine kinase inhibitor, has been shown to promote ferroptosis and to inhibit brain metastasis in a novel syngeneic model of spontaneous human epidermal growth factor receptor 2-positive breast cancer metastasis [122]. These data further support the role of ferroptosis in inhibiting tumor metastasis.

Emerging evidence has suggested that ferroptosis is an inhibitor of prognosis and overall survival in clinical. The most direct evidence is that SLC7A11 has been shown to be significantly elevated in RCC. Specifically, as an independent predictive factor, SLC7A11 overexpression is associated with a poor clinical prognosis [116]. ATP-binding cassette subfamily C member 5 (ABCC5) also has been found to be upregulated in HCC clinical samples and negatively correlated with ferroptosis by increasing the intracellular GSH levels and stabilizing SLC7A11. More importantly, HCC cells resistant to sorafenib have shown a substantial increase in ABCC5 expression, which is strongly associated with a worse prognosis [123, 124]. In addition, it has reported been reported that ACSL4 and GPX4 regulate ferroptosis in opposite ways. Unlike GPX4, which has been demonstrated to be downregulated in breast cancer cells, ACSL4 has been verified to be substantially expressed in breast cancer tissues [125, 126]. Moreover, ACSL4 and GPX4 expression, alone or combined, can be used as an independent prognostic factor for disease-free survival. Patients with high ACSL4 expression have a better overall survival, while a higher GPX4 expression is associated with a better distant metastasis-free survival [127]. Taken together, the above studies suggest that ferroptosis can act as a tumor suppressor through different molecular and cellular mechanisms.

### Ferroptosis as a tumor activator

Previous studies have indicated that GPX4 inactivation and ferroptosis caused by iron accumulation are important factors in tumor suppression. However, recent studies suggest that ferroptosis may function as a potential carcinogen under special conditions. For example, knockdown of *Gpx4* or a high-iron diet significantly increased the pancreatic weight, pancreatic intraepithelial neoplasia formation, and stromal response as well as increased the mortality in Kirsten rat sarcoma viral oncogene homolog (Kras)4-driven animals. Nevertheless, the number of typical acinar cells was drastically diminished. More importantly, tumor metastases to the liver and lungs were most prevalent in 10–12-month Pdx1-Cre, Kras^G12D/+^, Gpx4^−/−^ mice. These results suggest that the lack of *Gpx4* or a high-iron diet speeds up the development of pancreatic ductal adenocarcinoma induced by *Kras* [128]. Moreover, Ma et al. have shown that CD36 facilitates fatty acid absorption via tumor-infiltrating CD8^+^ T cells. In particular, the upregulation of CD36 reduces the antitumor activity of CD8^+^ T cells by inducing lipid peroxidation and ferroptosis. Of note, the antitumor ability of CD8^+^ T cells can be obviously renewed with CD36 deletion or suppression of ferroptosis [129]. However, the underlying regulatory mechanisms and specific pathways remain largely unknown.

## The prospect of ferroptosis in cancer therapy

Although ferroptosis plays a dual role in the development of tumors, it is typically considered as a well-known tumor suppressor in specific situations, as mentioned above. Due to the fact that ferroptosis inducers can be synthesized and have been verified in multiple in vivo experiments. Ferroptosis inducers have the potential to represent an anticancer therapy for clinical application after large-scale population validation. In line with this notion, numerous studies have developed different inducers to activate ferroptosis and explored the role of ferroptosis in different cancer therapies, such as such as chemotherapy, immunotherapy, radiotherapy, nanotherapy, SDT and PDT.

### Role of ferroptosis in chemotherapy

Chemotherapy is one of the main methods to treat cancer. Despite the clinical success of cancer chemotherapy, drug resistance continues to be a major and complex issue. Fortunately, some synthetic and natural compounds have demonstrated promising effects by activating ferroptosis, effectively inhibiting acquired tumor drug resistance and optimizing cancer efficacy (summarized in Table 3).

**Table 3:** Synthetic and natural compounds regulating ferroptosis in cancer therapy.

Name	Molecular structure	Targets	Models	Tumor	Reference	Phase of clinical development
Sorafenib		GPX4 and SLC7A11	Huh7, PLC/PRF5, PANC-1, BxPC-3, HCT116, and HT-29 cell lines	Hepatocellular carcinoma; pancreatic adenocarcinoma; colon carcinoma	[124, 130, 131]	Approved
Cisplatin		GPX4	Nude mice; A549, NCI‐H460, and H1299 cell lines	Lung cancer	[132]	Approved
Erastin		ROS and iron	Xenograft models; DU145, PC3, 22Rv1, LNCaP, and NCI-H660 cell lines	Prostate cancer	[133]	Preclinical
RSL3		ROS and iron	Xenograft models; DU145, PC3, 22Rv1, LNCaP, and NCI-H660 cell lines	Prostate cancer	[133]	Preclinical
JQ1		GPX4 and system X_C_^—^	Nude mice; MDA-MB-231, Hs578T, and A549 cell lines	Breast cancer, lung squamous cell carcinoma	[136]	Preclinical
PdPT		GPX4	Nude mice; A549 and NCI-H1299 cell lines	Nonsmall cell lung cancer	[137]	Preclinical
Lidocaine		SLC7A11	Patient tumor tissues; nude mice; SKOV-3 and T47D cell lines	Ovarian and breast cancer	[138]	Preclinical
Ketamine		GPX4	Patient tumor tissues; BALB/c nude mice; HepG2 and Huh7 cell lines	Liver cancer	[67]	Approved
MMRi62		P53	Patient tumor tissues, Panc1, BxPc3, panc10.05, AsPc1, Capan2, and SW1990 cell lines	Pancreatic cancer	[141]	Preclinical
Bupivacaine		GPX4 and SLC7A11	Nude mice; T24 and 5637 cell lines	Bladder cancer	[139]	Approved
6-Gingerol (natural)		USP14 and GPX4	Nude mice; A549 cell line	Lung cancer	[94]	Preclinical
DMOCPTL (natural)		GPX4	Bar b/c mice; MDAMB-231, SUM159, BT574, and 4T1 (mouse) cell lines	Triple-negative breast cancer	[146]	Preclinical
Bufotalin (natural)		GPX4	BALB/c nude mice; A549 cell line	Nonsmall cell lung cancer	[147]	Preclinical
Alloimpertorin (natural)		GPX4 and SLC7A11	MCF-10A, MDA-MB-231, and MCF-7 cell lines	Breast cancer	[149]	Preclinical

*GPX4* glutathione peroxidase 4, *ROS* reactive oxygen species, *SLC7A11* solute carrier family 7 member 11, *system XC*—, cystine-glutamate antiporter, *USP14* ubiquitin-specific protease 14.

#### Synthetic compounds

At present, the classic activators of ferroptosis mainly include sorafenib and cisplatin, which have been used clinically, and some preclinical experimental compounds such as erastin and RSL3. Among them, sorafenib is the first drug approved for the treatment of HCC and RCC. However, recent research regarding pancreatic adenocarcinoma and colon carcinoma have shown that the anticancer activity of sorafenib mainly depends on the inhibition of SLC7A11/GPX4 activity to induce ferroptosis; the activation of ferroptosis in turn promotes the anticancer effect of sorafenib [124, 130, 131]. On the other hand, cisplatin, one of the best metal-based chemotherapeutic drugs [132], has been demonstrated to play a non-negligible role in the regulation of lung cancer progression via targeting ferroptosis. Mechanistically, invasion and migration assays have shown that the combination of cisplatin and RSL3 can suppress the invasion and migration of H1299 and A549 cells more effectively. Moreover, inhibition of GPX4 by RSL3 has been reported to markedly enhance the anticancer effects of cisplatin by inhibiting tumor growth [99]. In addition, the activity of erastin and RSL3 has been used as a single or combined therapy for prostate cancer [133]. Ferroptosis induced by erastin and RSL3 not only inhibits cell migration and invasion, but it also delays the growth of drug-resistant prostate cancer tumors. It is worth noting that this treatment did not cause side effects such as weight loss or pain in mice [133].

In view of the complex antitumor effect of ferroptosis, more types of ferroptosis inducers have been actively developed. For example, the selective small molecule inhibitor, (+)-JQ1(JQ1) is the first potent and selective inhibitor of the bromodomain-containing protein 4 signaling pathway that has been reported to inhibit the proliferation of esophageal squamous cell carcinoma [134] and multiple myeloma [135]. In addition, Sui et al. have revealed that JQ1 induces ferroptosis by increasing ROS levels in breast cancer and lung squamous cell carcinoma cells. The rate of cell death was dramatically accelerated when JQ1 was coupled with the ferroptosis inducer RSL3. Further experiments have confirmed that the anticancer effect of JQ1 in breast cancer and lung squamous cell carcinoma cells can be enhanced by ferroptosis inducers [136]. Additionally, significant decreases in the tumor volume, size, and weight were observed after treatment with the broad spectrum deubiquitinase inhibitor palladium pyrithione complex (PdPT), and downregulation of GPX4 levels was detected, implying that PdPT may exert tumor-suppressing effects by relying on ferroptosis [137]. In line with these findings, the local anesthetic lidocaine has been proven to suppress the mRNA expression of SLC7A11 and to promote ferroptosis in ovarian and breast cancer cells in a dose-dependent manner, thereby repressing tumor growth [138]. Similarly, in bladder cancer, it has been shown that another widely used local anesthetic, bupivacaine, can inhibit the expression of SLC7A11 and GPX4 but increase the levels of Fe^2+^ and ROS, thus triggering ferroptosis and restraining the growth of xenografted tumors [139]. Furthermore, ketamine has been studied as rapid-acting anesthetic in cancer treatment [140]. A recent medical study has validated that ketamine can induce ferroptosis and limit cell proliferation, and its effect can be reversed by GPX4 overexpression in liver cancer [67]. Strikingly, emerging evidence has suggested that the small molecule MMRi62 can act as a novel ferroptosis inducer in pancreatic cancer; its use restricts tumor growth and metastasis by promoting the degradation of mutant p53 [141]. These findings lay the foundation for the clinical application of novel ferroptosis inducers and also encouraged us to explore whether additional ferroptosis inducers can cure cancer in an independent way. It would also be very interesting to test the combined effects of ferroptosis inducers with other existing anticancer drugs.

#### Natural compounds

Compared to traditional cancer therapies and drugs, natural compounds have shown unique advantages. Most importantly, they have attracted more and more attention because of their multiple benefits with minimal side effects [142]. Several studies have shown that 6-gingerol, a natural phenol in ginger (*Zingiber officinale* Roscoe), has potent anti-inflammatory, antitumor, and antioxidant effects [143–145]. It also has been demonstrated that 6-gingerol can significantly increase ROS and iron concentrations in vitro and in vivo, decrease the survival and proliferation of lung cancer cells, and reduce the tumor volume and weight in nude mice [94]. In triple-negative breast cancer, a derivative of the natural product parthenolide (DMOCPTL) has been reported to increase ROS accumulation in a time- and dose-dependent manner by directly targeting GPX4. Of note, DMOCPTL can significantly inhibit tumor growth and prolong the survival of mice, and it has no obvious toxicity [146]. Further research has demonstrated that a bufadienolide called BT, which can be isolated from the plant *Venenum bufonis*, exerts a similar effect. Specifically, BT has been shown to increase intracellular Fe^2+^ in vitro. In a xenograft model, BT inhibited tumor growth without significantly changing the mouse weight, downregulated GPX4, and induced lipid peroxidation, further supporting that BT exerts a tumor-suppressing effect with fewer side effects [147]. Besides, a recent study has shown that the natural coumarin alloimperatorin, found in the extract of *Angelica dahurica*, has anticancer properties [148]. Functionally, alloimperatorin directly decreases the expression levels of SLC7A11 and GPX4, thereby inhibiting the growth and invasion of breast cancer cells [149]. However, to date, there is still no direct clinical evidence of its therapeutic effect on human cancer. Therefore, in-depth research is required to determine whether these natural extracts have broad-spectrum effects, few side effects, and therapeutic effectiveness against human cancer.

### Role of ferroptosis in radiotherapy

Radiotherapy leads to the accumulation of ROS via the radiolysis of cellular water, which damages biomolecules including lipids; therefore, it is speculated that radiotherapy may be related to ferroptosis. To explore this possibility, Lang et al. performed staining with C11BODIPY, a lipophilic redox-sensitive dye, in HT1080 cells and found that radiotherapy resulted in an increase in lipid ROS concentrations. Furthermore, deletion of the ferroptosis-promoting gene *ACSL4* reduced the efficacy of radiotherapy. Most importantly, ferroptosis-resistant tumors remained radioresistant in vivo even at higher concentrations of radiotherapy [150]. These effects also have been confirmed in subsequent experiments. For example, data from The Cancer Genome Atlas have revealed that *SLC7A11* plays a role in the radioresistance of gliomas [151]. Additional experiments have demonstrated that the systemic X_C_^—^ inhibitor imidazole ketone erastin and RSL3 enhance the radiosensitivity of cancer cells through lipid peroxidation. The combined treatment with imidazole ketone erastin and sorafenib also has been reported to promote the inhibitory effect of radiation on tumor growth in a xenografted mouse model of sarcoma [151]. In line with these findings, Lei et al. have shown that ionizing radiation (IR) not only induces ROS but also increases the expression of ACSL4. In addition, inhibition of ACSL4 has been demonstrated to largely abolish IR-induced ferroptosis and to enhance radio resistance. Notably, IR increases the expression of SLC7A11 and promotes radio resistance by suppressing ferroptosis. Moreover, the inhibition of SLC7A11 or GPX4 has been shown to enhance IR sensitivity in radioresistant cancer cells and xenografts [152]. Further studies have found that radiotherapy induces ferroptosis, which in turn is associated with a better response to radiotherapy and a longer survival in esophageal cancer patients [152]. Collectively, ferroptosis plays a crucial role in radiotherapy-mediated tumor suppression; thus, inducing ferroptosis in radiotherapy-resistant tumors is a promising strategy for radio sensitization. However, for ferroptosis-resistant tumors that may also be radioresistant, ways to minimize their dual drug resistance must be discovered.

### Role of ferroptosis in immunotherapy

Immunotherapy is a new treatment method following surgery, radiotherapy, and chemotherapy, which brings the possibility of eradicating cancer. However, the immune escape is a major factor leading to the poor efficacy of tumor immunotherapy and has become a major obstacle of this method. Therefore, blocking the immune escape is one of the key issues to improve the effect of tumor immunotherapy. Ferroptosis has been reported to suppress tumor growth and metastasis via immune checkpoints. Previous studies have predicted that the immune checkpoint molecule programmed death-1 is positively correlated with the expression level of ACSL4 but negatively correlated with the expression levels of GPX4 and HSPB1 in clear cell renal cell carcinoma [153]. In line with this possibility, Liao et al. have demonstrated an increased overall survival or progression-free survival in patients with high ACSL4 expression following immune checkpoint blockade therapy [154]. Further experiments have indicated that BNP@R (including RSL3, PEG-*b*-P(DPA-*r*-PPa), and phenylboronic acid moieties grafted onto the mPEG-*b*-P (DPA-*r*-GC backbone)) activate the T-cell immune response by inducing ferroptosis in melanoma. Treatment combined with BNP@R and GPX4 inhibition has been shown to significantly induce programmed death-ligand 1 (PD-L1) expression both in vitro and in vivo. More importantly, combination treatment of BNP@R + laser and anti-PD-L1 induces ROS accumulation, which in turn significantly suppresses tumor growth and prolongs survival in mice [155]. More convincing evidence is that PD-L1 blockade treatment directly led to an increase of ROS in CD45-ID8 cells and effectively reduced tumor growth [156]. These data further support that tumor immune checkpoint inhibitor therapy based on ferroptosis is expected to provide a new strategy for tumor immunotherapy.

Besides, recent studies also have shown that ferroptosis can inhibit tumor cell immune escape via activating tumor immune cells, such as macrophages, natural killer cells, and T cells. To explore the effect of GPX4 on T cell physiological responses, Matsushita et al. constructed T cell-specific *Gpx4*-deficient mice and found that neither antigen-specific CD8^+^ nor CD4^+^ T cells lacking Gpx4 could expand and protect them from infection and that *Gpx4*-deficient T cells rapidly accumulated membrane lipid peroxides [157]. Additional studies have elucidated that CD8^+^ T cell-derived interferon gamma increases the binding of signal transducer and activator of transcription 1 (STAT1) to the transcription start site of SLC7A11 and inhibits its transcription. Thus, loss of STAT1 in tumor cells abrogates interferon gamma-mediated downregulation of SLC7A11 while reversing RSL3-induced lipid peroxidation and ferroptosis [156].

Macrophages are important immune cells in the human body and are mainly divided into M1 macrophages (typically activated or proinflammatory macrophages) and M2 macrophages (alternatively activated or anti-inflammatory macrophages) [158]. In general, M1 macrophages can generate ROS through the NADPH oxidase 2 pathway to help kill pathogens during immunity. Advanced glycation end products can induce M1 polarization in macrophages by stimulating the ROS/toll-like receptor 4/STAT1 pathway [159]. Moreover, iron overload-induced ROS generation and p53 acetylation also contribute to M1 polarization [160]. However, the levels of M1 markers such as interleukin-6, tumor necrosis factor-alpha, and interleukin-1beta can be increased by iron overload, while the levels of M2 markers are suppressed, thereby promoting the polarization of M1 macrophages [161]. This opens the door to the possibility of using M2 repolarization of tumor-associated macrophages as a method of tumor immunotherapy. Notably, it has been recently shown that M2 macrophages transform into M1 macrophages through the ferroptosis pathway, which can enhance anti-programmed cell death protein 1 immunotherapy in liver cancer [162]. In addition, bioinformatics analysis has revealed that high ferroptosis risk levels are closely associated with natural killer cell inactivation in breast cancer [163]. Moreover, in-depth research has determined that CAR-NK92MI cells can kill prostate cancer cells by inducing ferroptosis [164].

Collectively, ferroptosis has shown much promise in immunotherapy, and these findings have been confirmed in vivo and in vitro. Nonetheless, further prospective clinical trials are needed to validate its safety and efficacy as well as its precise mechanism.

### Role of ferroptosis in nanotherapy

Targeted drug delivery based on nanocarriers has the potential to improve the utilization rate of drugs, increase the intracellular uptake capacity of drugs, and reduce the toxic and side effects of drugs to a certain extent. Nanoparticles that are loaded with chemicals or biological components provide novel promise for enhancing the efficiency of existing inducers of ferroptosis and cancer therapy. Specifically, combination therapy of poly(ethylene glycol)-coated silica nanoparticles and amino acid starvation synergistically induce ferroptosis, and high-dose particle delivery can inhibit tumor growth and even lead to tumor regression [165]. Notably, the leakiness of the tumor vasculature is thought to be due to systemically injected nanoparticles accumulating in tumor tissue while causing nutrient deprivation within the tumor [166]. Interestingly, tumor blood vessels and nutrients are not destroyed when nanoparticles induce ferroptosis [165], laying the foundation for subsequent research. Another study has demonstrated that the antitumor activity of imidazole ketone erastin delivered in polyethylene glycol poly(lactic-co-glycolic acid) nanoparticles was enhanced in a mouse lymphoma model [167]. More recently, Li et al. have engineered a dual catalytic nanomedicine named Lipo-ART@CPNs (co-loading CuO_2_ nanodots and artemisinin) which can increase ROS levels, promote iron release, and lead to ferroptosis. Under the treatment of Lipo-ART@CPNs, the tumor inhibition rate of Lewis lung carcinoma-bearing mice was found to be increased by 18% compared to the non- Lipo-ART@CPNs treatment groups (58%). Furthermore, the tumor cells were obviously destroyed [168]. Consistent with this finding, copper nanodots (Cu NDs) that induce excess lipid peroxide and inhibit GPX4 and SLC7A11 to induce ferroptosis have been developed [169]. Similarly, in comparison to the control group, Cu NDs treatment group increased tumor growth inhibition to 90.8% after ultrasound (US). Most importantly, there were no statistically significant weight changes among all groups of mice, providing strong evidence for the safety and efficacy of this therapy [169]. Although further evidence is needed to support the superiority of this therapy, it will be interesting and promising to explore whether synergistic therapies combining ferroptosis and nanotherapy would be a more effective cancer treatment strategy.

### Role of ferroptosis in sonodynamic therapy

SDT is a treatment method that associates US sensitizer, oxygen with low intensity US to trigger ROS accumulation to kill cancer cells. Emerging evidence showed that ferroptosis combined with SDT has great clinical application potential due to its low cost, high penetration and non-invasive removal of solid tumors in a fixed way [170, 171]. Specifically, a previous report revealed that a specific sensitizer including Fe/Mn component could trigger ferroptosis in breast cancer under US [170]. One reason is that it can catalyze Fenton reaction to produce ROS, as well as increased the consumption of glutathione in cells. Of note, it can be clearly observed in mice that this treatment significantly inhibited the growth of tumor without affecting the blood index and weight of mice, which proved that the combination of ferroptosis and SDT can produce anti-tumor effect with minimal side effects [170]. Similar effects have also been verified in glioma. Zhu et al. developed a sensitizer called PIOC@CM NPs which combination of Fe3O4 and Ce6 was tested in vitro and in vivo [171]. The results showed that PIOC@CM NPs produced excessive ROS after US, which could not only reduce the cytotoxicity, but also increase the level of ACSL4. It is noteworthy that the introduction of ferroptosis and SDT significantly reduced the tumor volume and prolonged the survival of mice [171]. Further study constructed Aza-boron-dipyrromethene dyne (Aza-BDY, both an ferroptosis inducer and a sensitizer) and found it reduced cell viability after US [172]. Surprisingly, the synergistic effect of ferroptosis and SDT made the tumor inhibition rate reach 97.5%, and even eliminated the mice tumor completely [172]. Nevertheless, it is still unknown whether it can suppress recurrence and metastasis of cancer. Further in-depth research is required to determine whether ferroptosis combined with SDT has broad-range effects in different cancer cells and clinical efficacy.

### Role of ferroptosis in photodynamic therapy

PDT is a non-invasive treatment method that injects photosensitizer (PS) into the body, and then irradiates it at a specific wavelength to produce ROS, leading to oxidative stress and cell damage to treat tumor [173]. It exerts many effects combating different cancers, such as esophageal cancer, lung cancer and skin cancer [174–176]. However, due to the presence of intracellular antioxidants, especially GSH can effectively eliminate ROS, which seriously reduces the efficiency of PDT. To overcome this limitation, researchers introduced ferroptosis with PDT to enhance anti-tumor effect. A previous study conducted by Zhu et al. combined PS chlorine e6 (Ce6) and ferroptosis inducer erastin to form Ce6-erastin complex and verified in oral tongue square cell carcinoma (OTSCC) mice. Immunohistochemical analysis showed that the induction of PDT and ferroptosis decreased the expression of Ki-67 and significantly inhibited the expression of SLC7A11 compared with the control group [173]. Subsequently, further research developed PS called AE@RBC /Fe NCs triggered ferroptosis by reducing GSH level. Surprisingly, the participation of PDT further accelerated the consumption of GSH and increased the level of MDA. Of note, the tumor inhibition rate reached 95.8%, and the survival time of mice was significantly prolonged without affecting the liver and kidney functions of oral squamous cell carcinoma (OSCC) mice [177]. Furthermore, another PS named Fe3O4@Lipid NPs was recently reported to increase ROS level in a time and dose -dependent manner under irradiation in colorectal cancer. More importantly, it can be observed that the tumor was significantly necrotic, and the volume was significantly reduced without reducing the weight of mice [178]. These results supported the conclusion that the combination of ferroptosis and PDT has the superiority of anticancer effect. However, it remains to be explored whether this integration therapy has specificity and wide applicability in multiple cancers in terms of time, dose and irradiation wavelength.

## Conclusions and perspectives

Since its discovery in 2012, the role of ferroptosis in regulating a variety of cellular processes and different diseases, especially cancer, has been extensively studied [179, 180]. Ferroptosis plays a dual role in human tumorigenesis due to the complicated tumor microenvironment. Therefore, considering the tumor-suppressing effect of ferroptosis, the development of more specific inducers of ferroptosis may be a potential and promising cancer treatment strategy. In particular, due to the fact that various cancer cells have different sensitivities to ferroptosis, determining which cancers are more amenable to treatments incorporating ferroptosis will be an area of active research in the next few years. To date, only some classical compounds such as erastin, RSL3, etc. are more specific for ferroptosis, while other inducers, including sorafenib (the first line drug in unresectable or advanced HCC and RCC), are not specific for ferroptosis [81, 181]. With this concept in mind, it is necessary and urgent to screen and develop more specific activators of ferroptosis. On the other hand, using natural compounds or nanoparticles as ferroptosis inducers may be a safe and effective cancer treatment strategy due to their properties and few side effects. More importantly, combine ferroptosis inducers with other anticancer therapies will provide new sights for cancer treatment. With the exception of directly targeting ferroptosis, other approaches should be explored, such as the induction of ferroptosis through modulation of ncRNAs, transcription factors, and post-translational modifications (as summarized in Table 2). Given that ferroptosis is a double-edged sword in tumorigenesis, it will be interesting to explore the physiological role of ferroptosis in the progression of various cancers through conditional knockout or knockin mouse models. Going forward, cancer type-specific animal models of ferroptosis will facilitate and improve the development of specific ferroptosis promoters, and large-scale clinical trials will also help to accelerate their clinical translation. In the near future, it is believed that ferroptosis inducers with an optimal specificity and efficacy will be developed and used to treat various types of cancer.

## Supplementary information

aj-checklist

## Data Availability

All data in this study are available.
